# Comprehensive analysis including in‐game spending and violent game playing in patients with internet gaming disorder

**DOI:** 10.1002/npr2.12470

**Published:** 2024-07-28

**Authors:** Haruka Minami, Toshiyuki Shirai, Shohei Okada, Masao Miyachi, Takaki Tanifuji, Satoshi Okazaki, Tadasu Horai, Kentaro Mouri, Ikuo Otsuka, Akitoyo Hishimoto

**Affiliations:** ^1^ Department of Psychiatry Kobe University Graduate School of Medicine Kobe Japan

**Keywords:** addiction, adolescent, children, dependence, development

## Abstract

**Aim:**

Internet gaming disorder (IGD) is receiving increasing attention. In particular, violent gameplay or in‐game spending affects the psychiatric conditions and economic difficulties of patients. We conducted regression analysis and path analysis to investigate the associations between a comprehensive list of factors in patients with IGD, including the degree of internet or gaming dependence, developmental problems, family background, severity of depression, sleeping habits, in‐game spending, and first‐person shooter (FPS) and third‐person shooter (TPS) game playing.

**Methods:**

The participants were 47 Japanese individuals (39 males and 8 females) aged ≤20 years diagnosed with IGD with complete data from the internet addiction test, autism spectrum quotient, Quick Inventory of Depressive Symptomatology, and Pittsburgh Sleep Quality Index. All participants were asked whether their parents have divorce history, whether they have siblings, whether they play FPS or TPS games, and whether they engage in in‐game spending. Firstly, we compared these factors between males and females; secondly, we conducted regression analysis and path analysis in male patients.

**Results:**

As for simple comparison between sex, female patients showed greater severity of IGD and depressive score. In regression analysis of male patients, significant associations were found between FPS or TPS game playing and in‐game spending. We also created path diagrams.

**Conclusion:**

The results of the comprehensive analyses suggest the possibility that bidirectional synergistic effects could be achieved by gradually reducing both violent game playing and in‐game spending. The concept of internet dependence has a wide range of meanings, and for each subtype, it is important to consider the background that led to the dependence to make individualized environmental adjustments and provide psychotherapy.

## Abstract 2

We comprehensively analyzed factors in patients with internet gaming disorder (IGD) by multiple regression analysis and path analysis. In‐game spending and violent game playing may be synergistic. Female patients with IGD may be more likely to be depressed.

## Graphical abstract

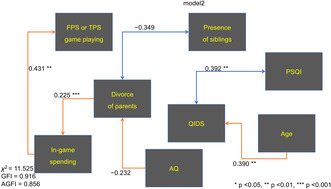

## 1 INTRODUCTION

Internet gaming disorder (IGD) has received increased attention in recent years. In particular, the prevalence of this disease has increased since the beginning of the COVID‐19 pandemic.^1^, ^2^ IGD is considered a lifestyle‐related disease and can cause brain dysfunction, especially in young people.^1^ Developmental problems and family backgrounds, such as divorce of the parents, have gradually been reported as factors in IGD.^3^, ^4^ Several studies on gaming have examined the impact of violent games on psychiatric symptoms and delinquency in young people.^5^, ^6^ Ivarsson et al. showed significant differences in pulse, post‐play emotional responses, and sleep quality in adolescents who habitually played games with more violent elements compared with those who played less violent games.^7^ In particular, first‐person shooter (FPS) and third‐person shooter (TPS) games contain much violent contents.^8^, ^9^ Furthermore, in recent years, the number of users of social games played via the internet has increased and excessive in‐game spending has become a problem. Uncontrollable in‐game purchases can result in high costs. Naturally, patients with IGD are expected to incur higher game costs.^10^ Both gaming disorder and gambling disorder are similar in that they have both been noted to have an impact on executive function, particularly on working memory.^11^ This could explain the tendency of patients with gaming dependence to engage in excessive in‐game spending. Psychiatric symptom aggravation caused by exposure to violent content and costly spending are likely to occur as secondary problems in patients with IGD.

There are various factors or consequences of internet gaming dependence, including developmental problems and depressive conditions,^3^, ^12^ and it is also fraught with secondary problems such as exposure to violence and increased in‐game purchases. However, few studies have comprehensively examined the associations between factors such as developmental problems, family background, psychiatric conditions, dependence on internet gaming, violence, and in‐game spending. We performed comprehensive analyses, including regression analysis and path analysis^13^, ^14^ to concisely show and organize comprehensive associations of various factors affecting IGD.

## 2 METHODS

### 2.1 Study sample

This study was approved by the Ethics Committee for Genetic Studies of the Kobe University Graduate School of Medicine. This retrospective study included patients aged ≤20 years who visited the outpatient clinic for internet gaming dependence at the Department of Psychiatry and Neurology, Kobe University Hospital between June 2021 and April 2023. Over this approximately 2‐year period, the number of patients aged ≤20 years, who received clinically four psychological examinations (internet addiction test [IAT], autism spectrum quotient [AQ], Quick Inventory of Depressive Symptomatology [QIDS], and Pittsburgh Sleep Quality Index [PSQI]), and diagnosed as IGD, was 47 (39 males and 8 females). The diagnoses of IGD were conducted by two or more skilled psychiatrists using the Diagnostic and Statistical Manual of Mental Disorders, 5th edition (DSM5). We asked all participants whether they play FPS or TPS games and whether they currently engage in in‐game spending (There are no set criteria for cost or last spending date). For FPS or TPS games, we presented APEX Legends, Call of Duty, Fortnite, PUBG BATTLEGROUNDS, and Wilderness Action as examples. Information on age, sex, FPS, or TPS game playing and in‐game spending was obtained from all participants. Furthermore, we asked all of them whether their parents had divorced and whether they had any siblings. All the participants were of Japanese descent. As this was a retrospective study, participants were given an informational document and offered the opportunity to be excluded from the study. After making sure there were no refusals, we included these 47 individuals in this study.

### 2.2 Psychological examinations

We obtained the results of four psychological examinations (IAT, AQ, QIDS, and PSQI) from all participants. The IAT consists of 20 questions scored on a five‐point Likert scale to measure dependency on the internet and games.^15^ The AQ is a self‐administered instrument for measuring autistic characteristics.^16^ The QIDS consists of 16 items to measure depressive symptoms.^17^ The PSQI consists of 19 items evaluating seven dimensions: subjective sleep quality, sleep duration, drug use, sleep latency, sleep efficiency, sleep disturbances, and daytime dysfunction.^18^

### 2.3 Comparison between males and females

First, differences in sex were analyzed for each variable. Continuous variables (age, AQ, IAT, QIDS, and PSQI) were compared with the Mann–Whitney *U*‐test, and categorical variables (divorce history of parents, presence of siblings, in‐game spending, FPS or TPS playing) were compared with Fisher's exact test. Since the number of female participants was extremely small compared to males, the following comprehensive analyses were conducted only on the 39 male patients, as the analyses could be affected by the difference in the number of male and female participants. Each variable of 39 male patients was standardized before following analyses.

### 2.4 Regression analysis for male patients

Age, AQ, divorce history of parents, and presence of siblings were considered as backgrounds of patients and were used only as explanatory variables in the regression analysis. IAT, QIDS, PSQI, in‐game spending, and FPS or TPS playing were considered variables that indicate current conditions and could be either explanatory or objective variables. We conducted regression analyses for respective objective variables, with other variables as explanatory variables. For instance, a regression analysis used IAT as objective variable and Age, AQ, divorce history of parents, presence of siblings, QIDS, PSQI, in‐game spending, and FPS or TPS playing as explanatory variables.

### 2.5 Correlation analysis and path analysis for male patients

Age, AQ, divorce history of parents, presence of siblings, IAT, QIDS, and PSQI, in‐game spending, and FPS or TPS game playing obtained from all participants were subjected to the one‐to‐one correlation analyses. We conducted path analyses with particular attention to in‐game spending and FPS or TPS playing as the final objective variables. We manually selected relationships that showed absolute correlation coefficients greater than 0.2 and used the R package lavaan, with maximum likelihood estimation with robust standard errors and a Satorra‐Bentler scaled test statistic. Three models were created: a model in which in‐game spending and FPS or TPS playing interact (model 1), a model in which in‐game spending affects FPS or TPS playing (model 2), and a model in which FPS or TPS playing affects in‐game spending (model3). The accuracies of the results were verified using the chi‐square value, goodness‐of‐fit index (GFI), and adjusted GFI (AGFI).

### 2.6 Statistical analysis

Statistical analyses were conducted using R version 4.3.1. Spearman's method was used for correlation analyses. If necessary, we used dummy values for divorce history of parents, no = 1 and yes = 2. For the presence of siblings, no = 1 and yes = 2. For in‐game spending, no = 1, yes = 2. For playing FPS or TPS, no = 1 and yes = 2. To use the sem function of the lavaan package, we adopted maximum likelihood with robust standard errors method.

## 3 RESULTS

### 3.1 Comparison between males and females

The demographic data, the results of each psychological examination, and psychological diagnosis by sex are presented in Table 1. QIDS (*p* = 0.0206) and IAT (*p* = 0.0209) were significantly higher in female patients with IGD, and FPS or TPS playing (*p* = 0.0479) was significantly higher in male patients with IGD. Because overwhelming number of participants were male (39 males and 8 females), the following analysis was conducted only on the 39 male patients.

**TABLE 1:** Demographic data and results of psychological examinations in male and female patients with IGD.

	Male	Female	*p*‐value
Demographic data			
Number of participants	39	8	
Age (mean ± SD)	14.18 ± 2.44	14.00 ± 1.69	0.697^a^
Psychological examination	Mean ± SD		
IAT	54.33 ± 12.40	67.38 ± 14.15	**0.0209** ^a^
AQ	22.69 ± 5.87	20.75 ± 11.18	0.487^a^
QIDS	6.51 ± 4.81	13.62 ± 9.30	**0.0206** ^a^
PSQI	6.54 ± 3.37	8.25 ± 5.60	0.469^a^
Family background	*N*		
Divorce history of parents	no: 34, yes: 5	no: 8, yes: 0	0.571^b^
Presence of siblings	no: 8, yes: 31	no: 1, yes: 7	1^b^
Characteristics of gaming	*N*		
In‐game spending	no: 10, yes: 29	no: 5, yes: 3	0.0894^b^
FPS or TPS game playing	no: 17, yes: 22	no: 7, yes: 1	**0.0479** ^b^
Psychiatric diagnosis			
Internet gaming disorder	39/39	8/8	
Autism spectrum disorder	23/39	5/8	
Attention‐deficit hyperactivity disorder	4/39	0/8	
Adjustment disorder	2/39	2/8	
Obsessive compulsive disorder	1/39	0/8	

*Note*: *p* < 0.05 is shown in bold.

Abbreviations: AQ, autism spectrum quotient; IAT, internet addiction test; IGD, internet gaming disorder; PSQI, Pittsburgh Sleep Quality Index; QIDS, Quick Inventory of Depressive Symptomatology; SD, standard deviation.

^a^ The *p*‐values were calculated with Mann–Whitney *U*‐test.

^b^ The *p*‐values were calculated with Fisher's exact test.

### 3.2 Regression analysis for male patients

We investigated the associations by regression analyses for IAT, QIDS, PSQI, in‐game spending, and FPS or TPS playing as objective variables respectively, with other variables as explanatory variables. These results are listed in Table 2. In regression analyses, QIDS and age (*p* = 0.0182), QIDS and PSQI (*p* = 0.0209), and in‐game spending and FPS or TPS playing (*p* = 0.0234) showed significant associations.

**TABLE 2:** Results of regression analysis in male patients with IGD.

Objective variables	Explanation variables	Estimate coefficient	Standard error	*z*‐Value	*p*‐Value
IAT ~	Age	−0.2289	0.1943	−1.178	0.248
	Divorce history of parents	0.0218	0.2047	0.106	0.916
	Presence of siblings	−0.1484	0.1890	−0.785	0.439
	AQ	0.1433	0.1900	0.754	0.456
	QIDS	0.1111	0.2064	0.538	0.594
	PSQI	0.0314	0.2006	0.156	0.877
	In‐game spending	−0.0692	0.2007	−0.345	0.733
	FPS or TPS playing	0.1820	0.1997	0.911	0.370
QIDS ~	Age	0.3978	0.1592	2.498	**0.0182**
	Divorce history of parents	−0.0856	0.1795	−0.477	0.637
	Presence of siblings	0.1077	0.1669	0.645	0.524
	IAT	0.0861	0.1600	0.538	0.594
	AQ	−0.0287	0.1688	−0.170	0.866
	PSQI	0.3934	0.1614	2.437	**0.0209**
	In‐game spending	−0.0556	0.1767	−0.315	0.755
	FPS or TPS playing	−0.0065	0.1783	−0.036	0.971
PSQI ~	Age	−0.2587	0.1746	−1.482	0.149
	Divorce history of parents	−0.00536	0.1862	−0.029	0.977
	Presence of siblings	−0.04297	0.1735	−0.248	0.806
	IAT	0.02598	0.1660	0.156	0.877
	AQ	−0.1812	0.1713	−1.058	0.299
	QIDS	0.4201	0.1724	2.437	**0.0209**
	In‐game spending	−0.05972	0.1826	−0.327	0.746
	FPS or TPS playing	0.2202	0.1798	1.225	0.230
In‐game spending ~	Age	−0.0764	0.1800	−0.425	0.674
	Divorce history of parents	0.2160	0.1817	1.189	0.244
	Presence of siblings	0.0779	0.1728	0.451	0.655
	IAT	−0.0570	0.1655	−0.345	0.733
	AQ	0.1803	0.1710	1.054	0.300
	QIDS	−0.0592	0.1880	−0.315	0.755
	PSQI	−0.0595	0.1819	−0.327	0.746
	FPS or TPS playing	0.4026	0.1685	2.389	**0.0234**
FPS or TPS playing ~	Age	0.1326	0.1776	0.747	0.461
	Divorce history of parents	0.1145	0.1834	0.624	0.537
	Presence of siblings	0.0085	0.1721	0.050	0.961
	IAT	0.1479	0.1624	0.911	0.370
	AQ	0.09649	0.1720	0.561	0.579
	QIDS	−0.0068	0.1870	−0.036	0.971
	PSQI	0.2163	0.1766	1.225	0.230
	In‐game spending	0.3970	0.1662	2.389	**0.0234**

*Note*: The coefficients are standardized. df indicates the degrees of freedom. *z‐*values indicate statistical significance. *p* < 0.05 is shown in bolded.

Abbreviations: AQ, autism spectrum quotient; FPS, first‐person shooter; IAT, internet addiction test; IGD, internet gaming disorder; PSQI, Pittsburgh Sleep Quality Index; QIDS, Quick Inventory of Depressive Symptomatology; TPS, third‐person shooter.

### 3.3 Correlation analysis and path analysis for male patients

The results of the correlation analyses are displayed in Table S1. No correlation showed significance after the post hoc test. When creating the path models, the location of the arrows between variables was the relationships where the absolute values of the correlation coefficients were greater than 0.2. In‐game spending and FPS or TPS playing were preferentially used as objective variables. Age was considered to affect QIDS, AQ was considered to affect divorce history of parents, and divorce history of parents was considered to affect in‐game spending. Divorce history of parents and presence of siblings were considered to interact, and QIDS and PSQI were considered to interact each other. In Model 1, in‐game spending and FPS or TPS playing were considered to interact, in Model 2, in‐game spending affects FPS or TPS playing, and in model3, FPS or TPS playing affects in‐game spending.

**FIGURE 1:**
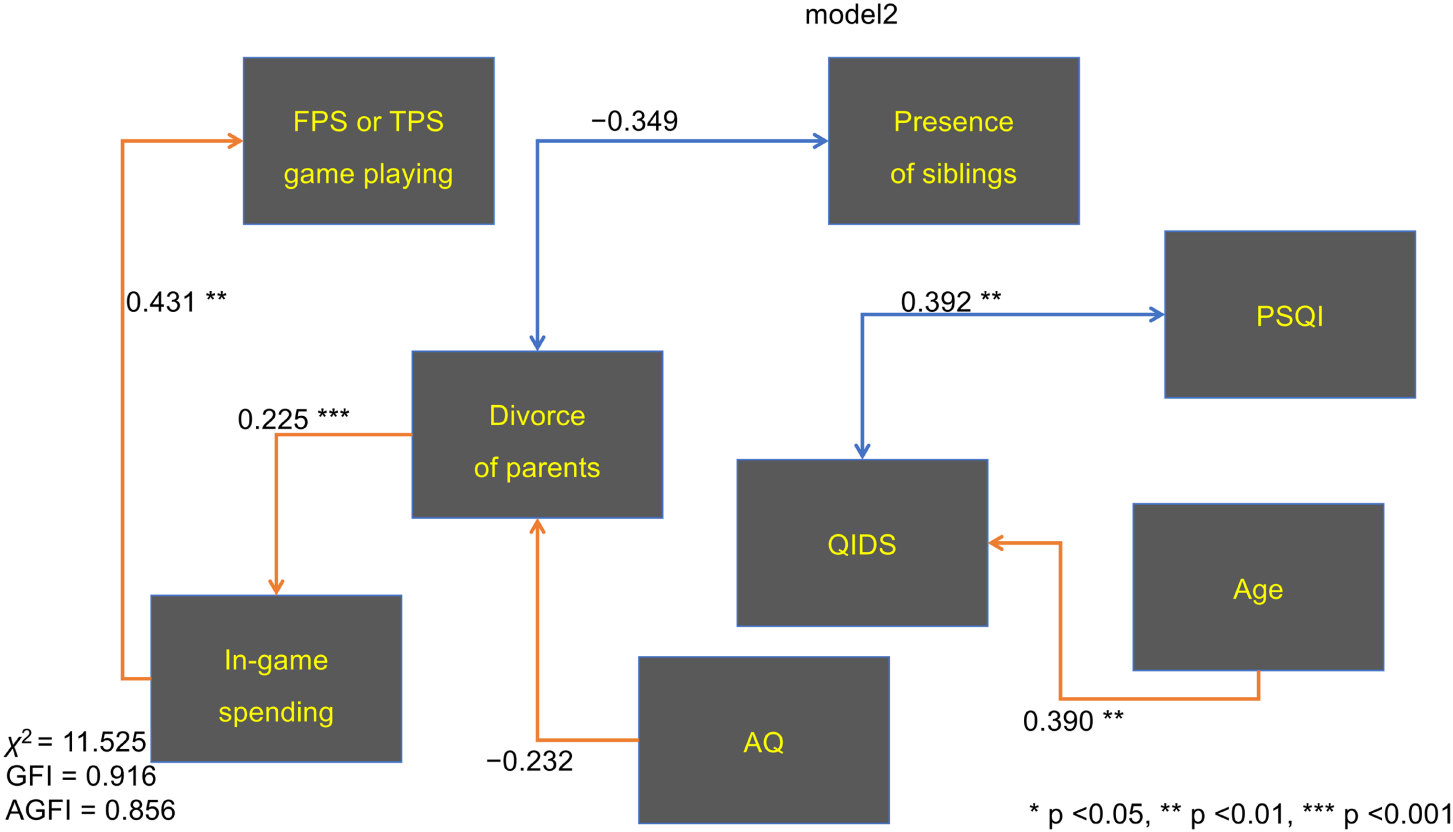
Path analysis diagram for Model 2. The red arrows with heads on one side indicate regression, blue with heads on both sides indicate interaction. The numbers next to arrows shown standardized regression coefficients for regression and covariance for interaction. AGFI, adjusted goodness‐of‐fit index; AQ, autism spectrum quotient; FPS, first‐person shooter; GFI, goodness‐of‐fit index; IAT, internet addiction test; PSQI, Pittsburgh Sleep Quality Index; QIDS, Quick Inventory of Depressive Symptomatology; TPS, third‐person shooter. *p < 0.05, **p < 0.01, ***p < 0.001.

The results of the path analyses are shown in Figure S1 for Model 1 (AGFI = 0.845, GFI = 0.909, and *χ* ^2^ = 12.476), Figure 1 for Model 2 (AGFI = 0.856, GFI = 0.916, and *χ* ^2^ = 11.525), and Figure S2 for Model 3 (AGFI = 0.779, GFI = 0.894, and *χ* ^2^ = 11.839). Model 2 was judged to fit best in the three models. Details of the regressions and correlations between the variables are listed in Table S2 for Model 1, Table S3 for Model 2, and Table S4 for Model 3.

## 4 DISCUSSION

To the best of our knowledge, this is the first study to perform comprehensive analyses using the results of several psychological examinations, including the IAT, with divorce history of parents, presence of siblings, in‐game spending, and FPS or TPS game playing.

In‐game spending and FPS or TPS game playing showed significantly associated in regression models. Although there is literature on the themes of in‐game spending or violent game playing individually, we could not find any studies that directly examine the impacts of both. The inability of adolescents to make appropriate in‐game purchases is due to immature neurological development, difficulty in controlling behavior, and poor planning.^19^, ^20^ The neurological features are closely associated with abnormalities in midbrain dopaminergic areas (reward processing areas) such as the ventral striatum and prefrontal cortex,^21^, ^22^ and includes overlap with brain regions associated with empathic abilities and those that play an important role in violent behavior.^23^ An association between FPS or TPS game playing and in‐game spending was found in this study, and it is possible that bidirectional synergistic effects could be achieved in gradually reducing both behaviors in treatment. However, we could not determine whether this would lead to the successful treatment of gaming dependence itself.

Attention‐deficit/hyperactivity disorder (ADHD), one of developmental problems, is often comorbid with various substance use disorders.^24^ ADHD is also a common background disorder for IGD, a behavioral dependence, which may co‐occur due to dysfunctions in the executive control network of patients with ADHD.^25^, ^26^ Conversely, autism spectrum tendency, another developmental problem, has also been reported to be related to game dependence, but there are fewer studies supporting this.^3^, ^27^ In this analysis, there was not significant associations between AQ and IAT. Children with autism spectrum disorder have limited interests and difficulties with reciprocal communication^28^ and tend to find their place online. The global COVID‐19 pandemic has significantly impacted these children.^29^ The measurement of autistic traits or diagnosis of ASD must be made by psychiatrists familiar with child and adolescent treatment using objective indices, such as the Autism Diagnostic Observation Schedule (ADOS) and Autism Diagnostic Interview‐Revised (ADI‐R),^28^, ^30^ which are rarely performed in daily practice in Japan. The AQ is self‐administered and cannot be a highly reliable indicator for children with ASD who are not very good at objectively assessing themselves.^31^, ^32^

As differences in sex, males were more likely to prefer violent games, and females were more likely to have higher QIDS and IAT score. Males with IGD are more likely to be influenced by violent content in games and engage in problematic behaviors.^33^, ^34^ It is natural to assume that they are more likely to prefer violent games. Females with IGD are more likely to have a history of depression.^35^, ^36^ The QIDS is a depression scale, and higher scores have been found in female IGD patients. However, the small number of females in this study requires caution in interpreting the results.

This study had several limitations. First, we used the results of self‐administered psychological examinations. Further research using more objective indices, such as the ADOS and ADI‐R for ASD, is needed. We did not consider the presence or absence of ASD. Second, we believe that the concept of IGD is too broad to be considered as a single dependence. The type of internet dependence may vary, such as gaming, use of the internet in general, or use of social networking services. Third, the sample size for this study was very small, especially among females, although it included comprehensively patients who visited outpatient Internet/gaming dependence. The results of studies with larger sample size need to be confirmed. Fourth, we could not obtain information such as the patient's intelligence quotient or the income of the parents.

## 5 CONCLUSION

Despite these limitations, we believe that this study provides a comprehensive overview of the associations between internet gaming dependence, developmental characteristics, mental state, family backgrounds, in‐game spending, and FPS or TPS game playing, as indicated by regression analysis and illustrated by path analysis. This study suggests that FPS or TPS game playing and in‐game spending may be synergistic and that female patients with IGD are more likely to experience severe levels of internet dependence and depression. In the future, detailed observations of the background and components that lead to each subtype of IGD as well as individualized environmental adjustment and psychotherapy are necessary.

## AUTHOR CONTRIBUTIONS

Haruka Minami: Data curation; formal analysis; investigation; methodology; resources; writing—original draft. Toshiyuki Shirai: Data curation; formal analysis; investigation; methodology; visualization; writing—original draft. Shohei Okada: Formal analysis; investigation; methodology; resources. Masao Miyachi: Data curation; formal analysis; investigation; methodology. Takaki Tanifuji: Conceptualization; methodology. Satoshi Okazaki: Investigation; methodology. Tadasu Horai: Data curation; formal analysis. Kentaro Mouri: Data curation; formal analysis. Ikuo Otsuka: Conceptualization; supervision. Akitoyo Hishimoto: Conceptualization; supervision; writing—review and editing.

## FUNDING INFORMATION

This study was supported by the JST (Moonshot R&D Program) grant number [JPMJMS239F] (Akitoyo Hishimoto) and JSPS KAKENHI [grant number JP21K07545 (Ikuo Otsuka)]. We thank Masako Kuranaga and Naoko Iwamoto for their assistance with the psychological examinations.

## CONFLICT OF INTEREST STATEMENT

The authors declare no conflict of interest.

## ETHICS STATEMENT

Approval of the Research Protocol by an Institutional Review Board: This study design and related procedures were performed in accordance with the Declaration of Helsinki. This study was approved by the Ethical Committee for Genetic Studies of Kobe University Graduate School of Medicine.

Informed Consent: As this was a retrospective study, participants were given an informational document and offered the opportunity to be excluded from the study.

Registry and the Registration No. of the Study: N/A.

Animal Studies: N/A.

## Supporting information


Appendix S1.

Appendix S2.

## Data Availability

The data supporting the findings of this study are available within the article and Appendix S2. The raw data table is in Appendix S1.
