# Supplementary material for: Comprehensive analysis including in‐game spending and violent game playing in patients with internet gaming disorder
Source: Neuropsychopharmacol Rep. 2024 Jul 28;44(3):631–8. doi: 10.1002/npr2.12470 (PMC11544446; doi:10.1002/npr2.12470)
Supplement: Supplementary file 2 — Appendix S2. [file NPR2-44-631-s002.docx]

***Supplementary Materials***

**Comprehensive analysis including in-game spending and violent game playing in patients with internet gaming disorder**

Haruka Minami*, Toshiyuki Shirai*, Shohei Okada, Masao Miyachi, Takaki Tanifuji, Satoshi Okazaki, Tadasu Horai, Kentaro Mouri, Ikuo Otsuka, Akitoyo Hishimoto

Department of Psychiatry, Kobe University Graduate School of Medicine, Kobe, Japan

*These authors contributed equally to this work

Correspondence: Ikuo Otsuka, M.D., Ph.D.

Department of Psychiatry, Kobe University Graduate School of Medicine

7-5-1 Kusunoki-cho, Chuo-ku, Kobe 650-0017, Japan

Tel.: +81-78-382-6065 Fax: +81-78-382-6079

Email address: otsuka19@med.kobe-u.ac.jp

**Table S1.** Results of one-to-one standardized correlation analysis of multiple variables of male patients with IGD.

| Correlation coefficients (p values ^†^) | Age | Divorce history of parents | Presence of siblings | IAT | AQ | QIDS | PSQI | In-game spending | FPS or TPS game playing |
| --- | --- | --- | --- | --- | --- | --- | --- | --- | --- |
| Age | 1 | −0.12089  (1) | −0.09723  (1) | −0.15379  (1) | 0.03828  (1) | 0.38033  (0.609) | −0.12327  (1) | −0.10842  (1) | 0.03493  (1) |
| Divorce history of parents | −0.12089  (1) | 1 | −0.37501  (0.672) | 0.09553  (1) | −0.26963  (1) | −0.11987  (1) | 0.02053  (1) | 0.22519  (1) | 0.18243  (1) |
| Presence of siblings | −0.09723  (1) | −0.37501  (0.672) | 1 | −0.12993  (1) | 0.09325  (1) | 0.06522  (1) | −0.01983  (1) | −0.00746  (1) | −0.06239  (1) |
| IAT | −0.15379  (1) | 0.09553  (1) | −0.12993  (1) | 1 | 0.11014  (1) | −0.03214  (1) | 0.10645  (1) | 0.10971  (1) | 0.19091  (1) |
| AQ | 0.03828  (1) | −0.26963  (1) | 0.09325  (1) | 0.11014  (1) | 1 | −0.09676  (1) | −0.16345  (1) | 0.19076  (1) | 0.14497  (1) |
| QIDS | 0.38034  (0.609) | −0.11987  (1) | 0.06522  (1) | −0.03214  (1) | −0.09676  (1) | 1 | 0.27476  (1) | −0.13111  (1) | 0.07851  (1) |
| PSQI | −0.12327  (1) | 0.02053  (1) | −0.01983  (1) | 0.10645  (1) | −0.16345  (1) | 0.27476  (1) | 1 | −0.07073  (1) | 0.16148  (1) |
| In-game spending | −0.10842  (1) | 0.22519  (1) | −0.00746  (1) | 0.10971  (1) | 0.19076  (1) | −0.13111  (1) | −0.07073  (1) | 1 | 0.43118  (0.221) |
| FPS or TPS game playing | 0.03493  (1) | 0.18243  (1) | −0.06239  (1) | 0.19091  (1) | 0.14497  (1) | 0.07851  (1) | 0.16148  (1) | 0.43118  (0.221) | 1 |

These correlation coefficients were calculated by Spearman’s method, using standardized values.

^†^ The p values were calculated with correlation analyses and Turkey adjustment.

Abbreviations: AQ, Autism Spectrum Quotient; FPS, first-person shooter; IAT, Internet Addiction Test; IGD, Internet gaming disorder; PSQI, Pittsburgh Sleep Quality Index; QIDS, Quick Inventory of Depressive Symptomatology; TPS, third-person shooter

**Table S2.** Results of path analysis for the model1

|  | Estimate coefficient | Standard error | z value | p value |
| --- | --- | --- | --- | --- |
| Regressions: |  |  |  |  |
| Divorce history of parents ~ |  |  |  |  |
| AQ | −0.232 | 0.131 | -1.769 | 0.0769 |
| QIDS ~ |  |  |  |  |
| Age | 0.390 | 0.120 | 3.258 | 0.00112 |
| In-game spending ~ |  |  |  |  |
| Divorce history of parents | 0.152 | 0.074 | 2.045 | 0.0409 |
| Covariances: |  |  |  |  |
| Divorce history of parents ~~ Presence of siblings | −0.349 | 0.219 | -1.597 | 0.110 |
| QIDS ~~ PSQI | 0.392 | 0.147 | 2.662 | 0.00777 |
| In-game spending ~~ FPS or TPS playing | 0.393 | 0.149 | 2.638 | 0.00834 |
| Overall |  |  |  |  |
| RMSEA | 0.000 |  | | |
| df | 21 |  | | |
| *χ*^2^ | 12.476 |  | | |
| GFI | 0.909 |  | | |
| AGFI | 0.845 |  | | |

The coefficients are standardized coefficients. df indicates degree of freedom. The *z* values indicate statistic value.

Abbreviations: AGFI, adjusted goodness of fit index; AQ, Autism Spectrum Quotient; FPS, first-person shooter; GFI, goodness of fit index; PSQI, Pittsburgh Sleep Quality Index; QIDS, Quick Inventory of Depressive Symptomatology; RMSEA, root mean square error of approximation; TPS, third-person shooter.

**Table S3.** Results of path analysis for the model2

|  | Estimate coefficient | Standard error | z value | p value |
| --- | --- | --- | --- | --- |
| Regressions: |  |  |  |  |
| Divorce history of parents ~ |  |  |  |  |
| AQ | −0.232 | 0.131 | -1.769 | 0.076893 |
| QIDS ~ |  |  |  |  |
| Age | 0.390 | 0.120 | 3.258 | 0.00112 |
| In−game spending ~ |  |  |  |  |
| Divorce history of parents | 0.225 | 0.059 | 3.797 | 0.000147 |
| FPS or TPS playing ~ |  |  |  |  |
| In−game spending | 0.431 | 0.135 | 3.203 | 0.001359 |
| Covariances: |  |  |  |  |
| Divorce history of parents ~~ Presence of siblings | −0.349 | 0.219 | -1.597 | 0.110 |
| QIDS ~~ PSQI | 0.392 | 0.147 | 2.662 | 0.00777 |
| Overall |  |  |  |  |
| RMSEA | 0.000 |  | | |
| df | 21 |  | | |
| *χ*^2^ | 11.525 |  | | |
| GFI | 0.916 |  | | |
| AGFI | 0.856 |  | | |

The coefficients are standardized coefficients. df indicates degree of freedom. The *z* values indicate statistic value.

Abbreviations: AGFI, adjusted goodness of fit index; AQ, Autism Spectrum Quotient; FPS, first-person shooter; GFI, goodness of fit index; PSQI, Pittsburgh Sleep Quality Index; QIDS, Quick Inventory of Depressive Symptomatology; RMSEA, root mean square error of approximation; TPS, third-person shooter.

**Table S4.** Results of path analysis for the model3

|  | Estimate coefficient | Standard error | z value | p value |
| --- | --- | --- | --- | --- |
| Regressions: |  |  |  |  |
| Divorce history of parents ~ |  |  |  |  |
| AQ | −0.232 | 0.130 | -1.790 | 0.0734 |
| QIDS ~ |  |  |  |  |
| Age | 0.390 | 0.121 | 3.230 | 0.00124 |
| In-game spending ~ |  |  |  |  |
| FPS or TPS playing | 0.404 | 0.148 | 2.721 | 0.00651 |
| Divorce history of parents | 0.152 | 0.074 | 2.045 | 0.0409 |
| Covariances: |  |  |  |  |
| Divorce history of parents ~~ Presence of siblings | −0.349 | 0.219 | -1.595 | 0.111 |
| QIDS ~~ PSQI | 0.392 | 0.147 | 2.662 | 0.00777 |
| Overall |  |  |  |  |
| RMSEA | 0.000 |  | | |
| df | 19 |  | | |
| *χ*^2^ | 11.839 |  | | |
| GFI | 0.894 |  | | |
| AGFI | 0.779 |  | | |

The coefficients are standardized coefficients. df indicates degree of freedom. The *z* values indicate statistic value.

Abbreviations: AGFI, adjusted goodness of fit index; AQ, Autism Spectrum Quotient; FPS, first-person shooter; GFI, goodness of fit index; PSQI, Pittsburgh Sleep Quality Index; QIDS, Quick Inventory of Depressive Symptomatology; RMSEA, root mean square error of approximation; TPS, third-person shooter.


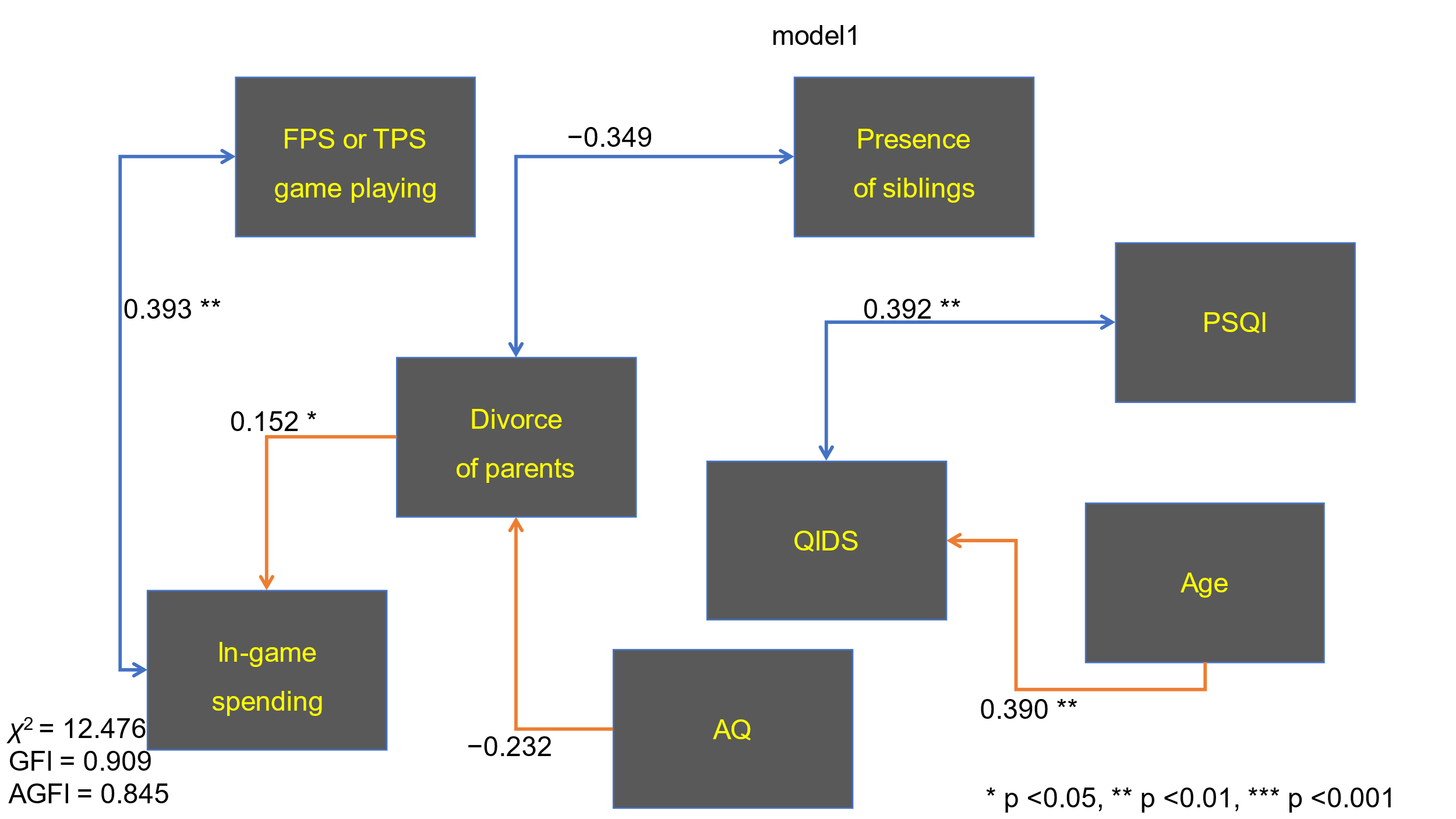


**Figure S1.** Path analysis diagram for model1.

The red arrows with heads on one side indicate regression, blue with heads on both sides indicate interaction. The numbers next to arrows shown standardized regression coefficients for regression and covariance for interaction.

Abbreviations: AGFI, adjusted goodness of fit index; AQ, Autism Spectrum Quotient; FPS, first-person shooter; GFI, goodness of fit index; IAT, Internet Addiction Test; PSQI, Pittsburgh Sleep Quality Index; QIDS, Quick Inventory of Depressive Symptomatology; TPS, third-person shooter.

* p < 0.05, ** p < 0.01, *** p < 0.001


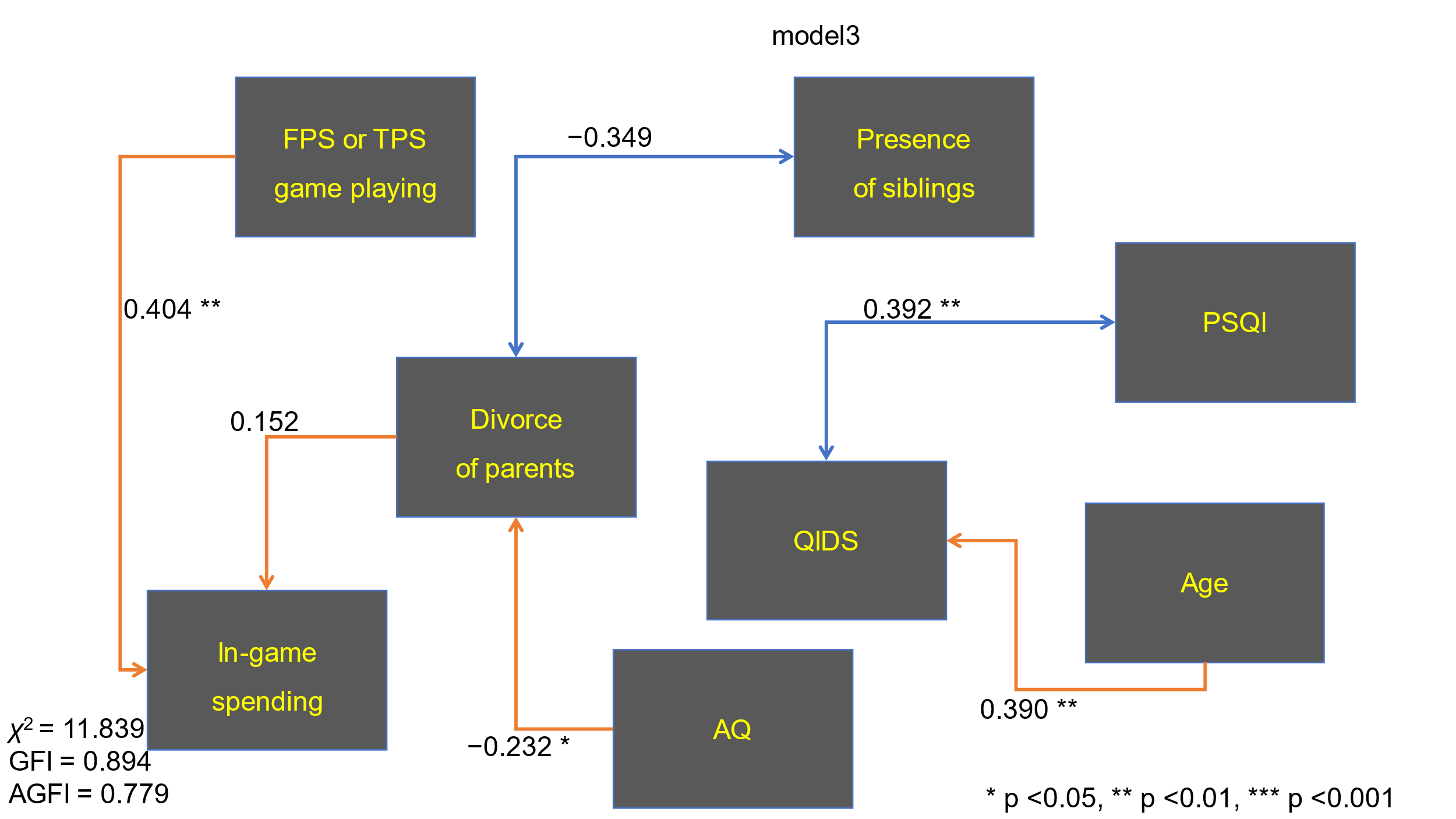


**Figure S2.** Path analysis diagram for model3.

The red arrows with heads on one side indicate regression, blue with heads on both sides indicate interaction. The numbers next to arrows shown standardized regression coefficients for regression and covariance for interaction.

Abbreviations: AGFI, adjusted goodness of fit index; AQ, Autism Spectrum Quotient; FPS, first-person shooter; GFI, goodness of fit index; IAT, Internet Addiction Test; PSQI, Pittsburgh Sleep Quality Index; QIDS, Quick Inventory of Depressive Symptomatology; TPS, third-person shooter.

* p< 0.05, ** p < 0.01, *** p < 0.001
